# ﻿Type designation and redescription of *Scolopendraspinosissima* Kraepelin, 1903 (Scolopendromorpha, Scolopendridae), with remarks on related taxa

**DOI:** 10.3897/zookeys.1215.129410

**Published:** 2024-10-17

**Authors:** Carles Doménech

**Affiliations:** 1 Departament de Ciències Ambientals i Recursos Naturals, Universitat d’Alacant, Carretera de Sant Vicent del Raspeig s/n C.P. 03690, San Vicent del Raspeig, Alacant, Spain Universitat d’Alacant Alacant Spain

**Keywords:** *Ethmostigmus*, *multidens*, *paradoxa*, Philippines, *rubripes*, *Scolopendra*, *spinosissima*

## Abstract

The recent description of the scolopendromorph centipede *Scolopendraparadoxa* Doménech, 2018 raised questions concerning the morphological limits of its closest relative *S.spinosissima* Kraepelin, 1903. Following the works of this author and other evidence, the specimens making up the type series of *S.spinosissima* and a lectotype are fixed, redescribed, and illustrated; these are then compared with this species’ unique available voucher and with *S.paradoxa* type material. Specimens making up the *S.spinosissima* type series are fixed including only four of the five individuals stored in the collection of Zoological Museum of Hamburg and, the voucher is identified as *S.spinosissima.* Its sister species, *S.paradoxa*, is confirmed as a morphologically and molecularly distinguishable taxon. Additionally, new data on the *S.spinosissima* type series are provideda and observations involving the excluded original type, reidentified as *Ethmostigmusrubripesrubripes* (Brandt, 1840), are given. Finally, the presence of *S.multidens* Newport, 1844 in the Philippines is proposed as dubious and a revised key for the *Scolopendra* of this archipelago is presented.

## ﻿Introduction

Centipedes, class Chilopoda, are one of the basal extant groups of terrestrial arthropods (Thomas et al. 2020). They are distributed across the world’s tropical, temperate, and subarctic areas, with their predatory activities occurring mostly at night. With a total of 3300 extinct and extant recognised taxa, centipedes are placed in five different orders (Edgecombe and Giribet 2007; Schileyko et al. 2020). One of the best-known of these is Scolopendromorpha, which accommodates the emblematic and quite diverse – with ~ 100 described species within (Bonato et al. 2016) – genus *Scolopendra* Linnaeus, 1758.

**Figure 1.**
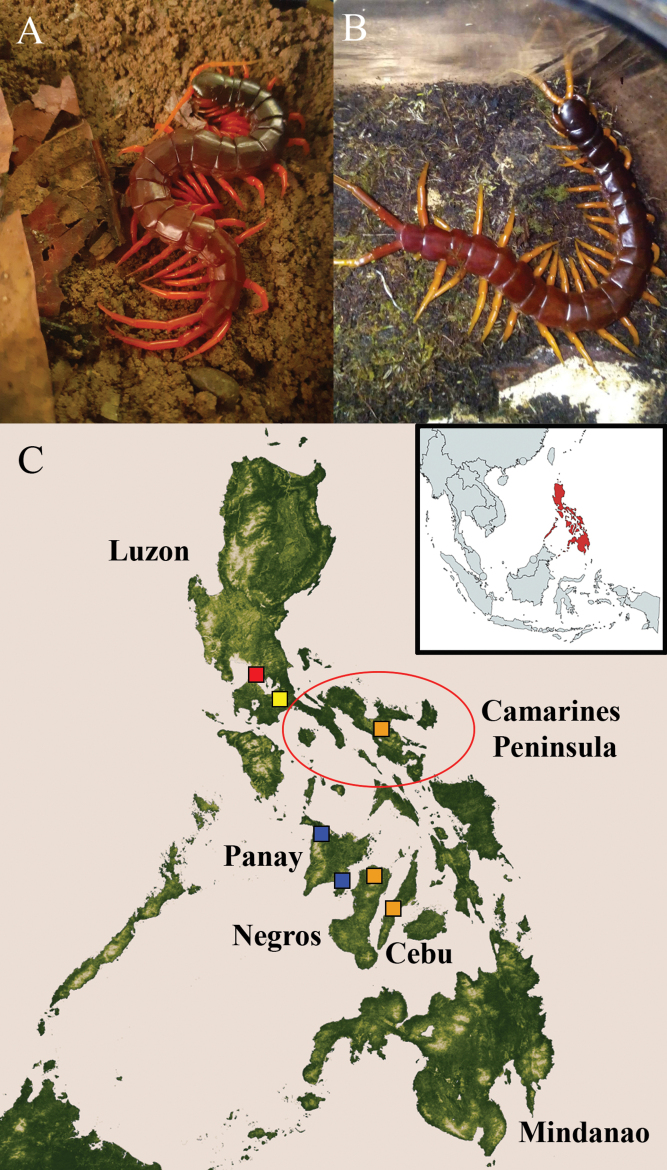
*Scolopendraspinosissima* Kraepelin, 1903 **A**, **B** habitus in vivo showing the only two known variations of colouration; specimens from Panay Is. (**A**) and Guimaras Is. (**B**) (photographs courtesy of E. Währen; individuals uncollected) **C** distribution, 20^th^ century records: red square = lectotype, Manila, Luzon Is.; red circle = paralectotypes 1–3, Camarines Peninsula, Luzon Is.; yellow square = non-types, Dolores, Quezon province (previously Tayabas), Luzon Is. (in the literature erroneously placed in Dolores, Camarines Peninsula, indicated by a red circle). 21^st^ century data: orange squares = (top) Mt. Isarog, Camarines Sur province, Luzon Is.; (middle) Cadiz, Negros Occidental province, Negros Is.; (bottom) Barili, Cebu Prov., Cebu Is. New data: blue squares: (top) Idiacacan, Pandan (Antique), Panay Is. (~ 11.683537; 122.114986); (bottom) Jordan, Guimaras Is. (~ 10.652871; 122.602054) (credits: E. Währen). Mindanao Is. indicates the origin of the specimen “Mus. Paris. I.XI.03” misidentified by Kraepelin as “*S.spinosissima*” (specimen currently excluded from the type series).

In the Philippines, the genus *Scolopendra* currently comprises six valid taxa, three of them endemic (Doménech et al. 2018). One of these, the reddish-brown *S.spinosissima* Kraepelin, 1903 (Fig. 1A, B), was initially described as a variety of *S.subspinipes* Leach, 1815. For the original description of this species, Kraepelin (1903) based the type series on an unspecified number of specimens stored at the Zoological Museum of Hamburg (ZMH collection, Germany), which were, in turn, on loan from the Natural History National Museum Paris (MNHN collection, France). Kraepelin (1903) did not indicate the exact type locality for the species, instead only assigning it to their origin country, Philippines. In 1904, a year after the description of this variety, this same taxonomist confirmed the presence of other *S.spinosissima* specimens in the MNHN collection (Kraepelin 1904b). In this catalogue, he also documented the sampling dates and localities, as well as names of collectors and previous examiners, but again he did not specify if these other specimens made up part of the type series.

In line with the remarks provided in Kraepelin’s original work (1903), Attems (1930) raised S.subspinipesvar.spinosissima to its current specific status. In that scrutiny, which was an almost literal transcription of Kraepelin’s (1903) text, a schematic illustration of an unidentified specimen of *S.spinosissima* was also added, detailing the ventral view of its ultimate leg-bearing segment and ultimate legs’ prefemora (Attems 1930: fig. 44; Doménech et al. 2018: fig. 14E).

Since Attems (1930), few reports on this species have been published. These citations were restricted to old distribution data (Wang 1951, 1962) (Fig. 1C), museum catalogues (Weidner 1960; Wang 1962; Rack 1974; Thofern et al. 2021), a dichotomous key (Lewis 2010), illustrations of its defensive behaviour (Kronmüller and Lewis 2015: fig. 2A), and recently, ethological notes (Acuña et al. 2021).

**Figure 2.**
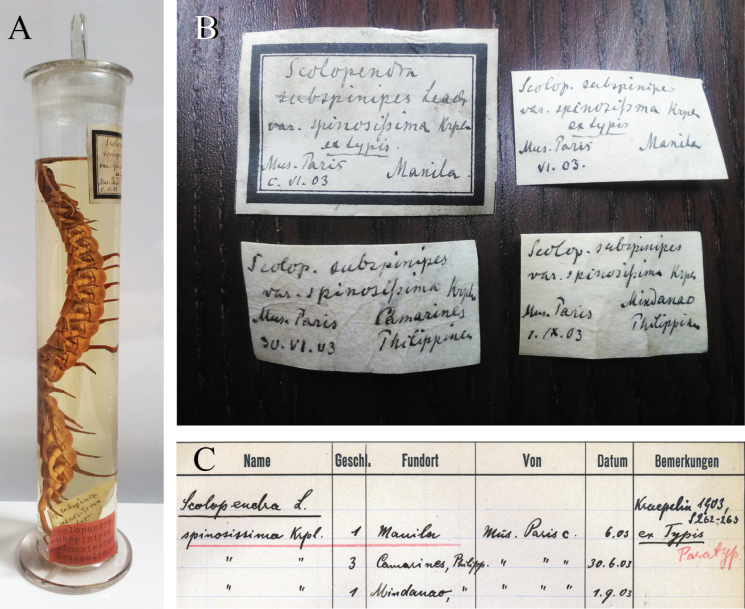
**A***Scolopendraspinosissima* Kraepelin, 1903; lectotype in original container (ZMH-A0000633). Notice the two labels inside and the red label fixed outside the jar, indicating type status **B** labels of the original type series proposed by Kraepelin in 1903: lectotype from Manila (two labels on top), paralectotypes from Camarines (bottom left), and specimen “Mus. Paris. I.XI.03” from Mindanao (bottom right, specimen excluded from the type series) **C** the manuscript list in which Kraepelin pointed out the type status of these specimens, localities, and identification dates. Notice that these five specimens, making up the type series, are ascribed to the data “S. 262–263”: S. is an abbrev. of “Seiten”, meaning pages in German, which is a reference to the page numbers of the 1903 publication containing the original description. Photograph courtesy of N. Dupérré.

Notwithstanding, it was not until the integrated description of *S.paradoxa* Doménech, 2018 that the morphological limits of *S.spinosissima* were questioned. The doubts regarding the morphology of the last-mentioned species derived from the ancient works of Kraepelin (1903) and Attems (1930), where some omitted, controversial, or unrestrictive data complicated these species differentiations. Inconsistent with the differences found at the genetic level (Doménech et al. 2018), it was noted that when the *S.paradoxa* type series was compared with the description of *S.spinosissima*, a few taxonomic traits of each species occasionally overlapped. This revealed that the existing morphological standard of *S.spinosissima* was inadequately defined, and as a result, the need for this species redescription emerged (Doménech et al. 2018).

To emend this situation, related literature, additional external sources (including museum catalogues and labels), and all probable specimens making up the *S.spinosissima* type series (Kraepelin 1903, 1904b; Thofern et al. 2021) were examined in detail. As a result, the specimens making up the type series of *S.spinosissima* were fixed and a lectotype was designated (Fig. 2A–C). The taxon was redescribed and illustrated before comparing it with the unique available voucher of this same species and with the *S.paradoxa* type material (ICZN 1999: Preamble, Art. 13, Recommendation 13A; Doménech et al. 2018; Buckner et al. 2021). Based on unexpected findings, some considerations regarding the distribution and taxonomy of two other Asian species of Scolopendridae are also briefly discussed, and a revised key for *Scolopendra* species from the Philippines is provided.

## ﻿Materials and methods

### ﻿Morphological and taxonomic analysis

The specimens from the collection of Zoological Museum of Hamburg (**ZMH**) and Colección Entomológica de la Universidad de Alicante (**CEUA**) were checked at the Universidad de Alicante (**UA**) under a Leica M205C stereo microscope, connected with a montage imaging system Leica DFC450 operated under the Cell’D program. Measurements were made with a Monzana® Digital Vernier Caliper. The specimens from Natural History National Museum in Paris (**MNHN**) collection were examined using a Wild Heerbrugg M3C stereomicroscope and photographed with a Nikon Coolpix P5100 digital camera.

Species identification and the proposed key were based on Newport (1844), Pocock (1898), Kraepelin (1903), Attems (1930), Schileyko and Stagl (2004), Lewis (2010), Kronmüller and Lewis (2015), Schileyko and Stoev (2016), Siriwut et al. (2016), Doménech et al. (2018), and Joshi and Edgecombe (2018). Sex determination and genitalia descriptions were based on the previous works of Demange and Richard (1969), Iorio (2003), Siriwut et al. (2016), and Doménech et al. (2018). Standardised terminology for centipede morphology follows Bonato et al. (2010). Labels and handwritten lists’ authorships were verified by comparing their typology and calligraphy with each other and those presented in Harms and Dupérré (2018), Monod et al. (2019), and Thofern et al. (2021). Labels were actualised following Wheeler et al. (2001). Throughout the text, the citation of the type series of the nominal taxon of S.subspinipesvar.spinosissima is replaced by the shorter and currently valid nomenclature *S.spinosissima.* Distribution maps have been created according to data from Kraepelin (1903, 1904b; also labels and notes), Attems (1930), Wang (1962), Lewis (2010), Doménech et al. (2018), Acuña et al. (2021), and iNaturalist (2022) database (https://www.inaturalist.org/observations/106848806 and https://www.inaturalist.org/observations/104968341; E. Währen pers. comm. April–May 2022). Fig. 1C was generated on the base maps obtained from the National Oceanic and Atmospheric Administration, National Weather Service (NOAA/NWS) website (www.noaa.gov; accessed Nov. 2022; reproduced and modified according to their licenses and personal instructions; 7 Dec. 2022) and Mapchart free software (https://www.mapchart.net). Finally, image modifications – background removal, contrast, brightness, notes, and references in the illustrations – were made using Adobe Photoshop CS6 software.

### ﻿Institutional abbreviations

**BMB-DENR** Biodiversity Management Bureau – Department of Environment and Natural Resources, Philippines

**CEUA** Colección Entomológica de la Universidad de Alicante (UA), San Vicent del Raspeig, Alacant, Spain

**MNHN** Natural History National Museum, Paris, France

**PAE** Philippines Association of Entomologists, Inc., Philippines

**PNM** Philippine National Museum, Philippines

**PNU** Philippine Normal University, Philippines

**WRD-DENR** Wildlife Resources Division - Department of Environment and Natural Resources, Philippines

**ZMH** Zoological Museum of Hamburg, Germany

### ﻿Morphological abbreviations

General morphology:

**AP** apical spine

**DM** dorso-median process

**LS** lateral spine

**M** median process

**S**, **SS** sternite/s

**SAP** subapical spine

**SP** spine on prefemoral corner process

**T**, **TT** tergite/s

**UL** ultimate legs

**ULBS** ultimate leg-bearing segment

**V** ventral process

**VL** ventro-lateral process

**VM** ventro-median process

Genital region:

**AV** anal valve

**LA** lamina adanalis

**LS** lamina subanalis

**SGS I** sternite of genital segment 1

**SGS II** sternite of genital segment 2

### ﻿Comparative, supporting, and other related materials

**ZMH** – **Philippines** • 1 unsexed *Ethmostigmusrubripesrubripes* (Brandt, 1840) [determined by Kraepelin as *S.spinosissima*]; Mindanao; 1902; H. W. Brölemann leg.; “ScolopendrasubspinipesLeach.var.spinosissima Krpln. Mus. Paris. I.XI.03- Mindanao Philippinen”, ZMH-A00016061 (see listed material of this institution below).

**ZMH other material**: Kraepelin’s handwritten ZMH catalogue (p.102) and draft (p. 92; pointing to the definitive condition of the catalogue); containing determination dates, localities, previous storage emplacements, type status for each specimen, and page numbers referencing the samples ascribed to Kraepelin’s (1903) original publication (Fig. 2C). Widner’s card files referencing the ZMH*S.spinosissima* specimens type status, Kraepelin’s handwritten catalogue and the number of the pages in which the species was described as well as its historical nomenclatural considerations, according to Kraepelin (1903) and Attems (1930).

**MNHN** – **Philippines** • 1 unsexed adult *S.paradoxa* [determined by Kraepelin as *S.spinosissima*]; Luzon Island, Manila. “Manille”; 1902; H. W. Brölemann leg.; “Léveillé an *multidens* Nwpt. [unreadable] DCCXXIX”, “ScolopendrasubspinipesLeachvar.spinosissima Krpl.”, “Muséum Paris. Manille. Coll H. Brölleman, 1902. ScolopendrasubspinipesLeachvar.spinosissima Krpl. Auct. dét. 1903. (N° 729) (Léveillé) [Mus. staff summit label]”; MNHN N° 387; • 7 adults, 3 subadults, unsexed *S.spinosissima*; Luzon Island, Tayabas [Currently Quezon Province], Dolores; same collection data as above; “Dolores Tayabas. I. Philippines. Pen. Camarines [sic.]; • ? multidens C[?]XXXIX. Eug. Simon”, “ScolopendrasubspinipesLeachvar.spinosissima Krpl.”, “Muséum Paris. I. Philippines. Pen. Camarines [sic.]. Dolores Tayabas. Coll H. Brölleman, 1902. ScolopendrasubspinipesLeachvar.spinosissima Krpl. Auct. dét. 1903. (N° 149) (Eug. Simon) [Mus. staff summit label]”; MNHN N° 388.

**CEUA** – **Philippines** • 1 unsexed *S.spinosissima*; DNA voucher specimen, non-type; GenBank: KY888682.1; remaining data ibid. Doménech et al. (2018) CEUA016-Mr0009; • 1 adult male, 1 adult female, and 10 subadult unsexed *S.paradoxa* (type series and DNA vouchers); remaining data ibid. Doménech et al. (2018); CEUA016-Mr0000 to Mr0008 and CEUA017-Mr0000 to Mr0002. These materials were originally described in Doménech et al. (2018) and deposited at CEUA. They will be transferred to a permanent repository in a yet undetermined Philippine public institution based on an agreement with BMD-DENR [Biodiversity Management Bureau; Office BMB202305107 – Department of Environment and Natural Resources (DENR)]; document signed on 18 October 2023 (https://bmb.gov.ph). The use of these specimens has been approved for research purposes, including molecular studies, with prior notification to DENR. The author of this article would like to expressly indicate that access to biological resources in the Philippines always requires appropriate research permits from the DENR.

## ﻿Results

### ﻿Systematics

**Order Scolopendromorpha Pocock, 1895**

**Family Scolopendridae Leach, 1814**

**Subfamily Scolopendrinae Leach, 1814**

**Tribe Scolopendrini Leach, 1814**

**Genus *Scolopendra* Linnaeus, 1758**

#### ﻿ Scolopendra spinosissima

Taxon classificationAnimaliaScolopendromorphaScolopendridae

Kraepelin, 1903

C7A1D79B-93A5-5F6C-AEEF-71D0D627DA9F

**Figure 3.**
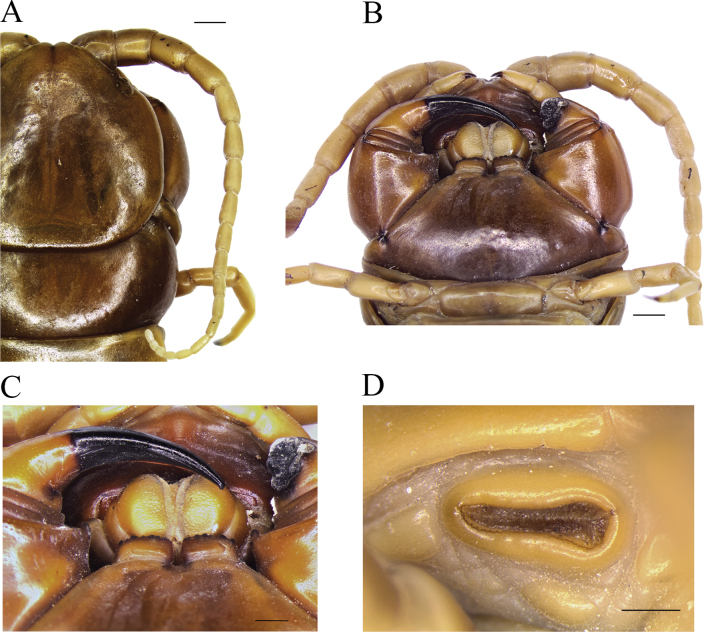
*Scolopendraspinosissima* Kraepelin, 1903; lectotype (ZMH-A0000633) **A** cephalic plate and right antenna, dorsal view **B** forcipular segment, ventral view **C** tooth-plates, ventral view **D** left spiracle on segment 8. Scale bars: 0.5 mm (**C, D**); 2 mm (**A, B**).

**Figure 4.**
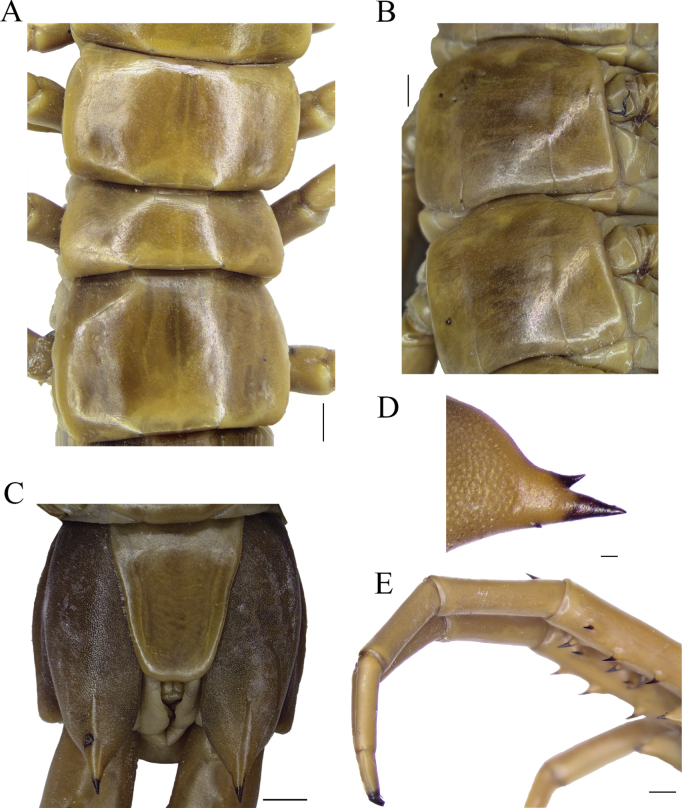
*Scolopendraspinosissima* Kraepelin, 1903; lectotype (ZMH-A0000633) **A** tergites 3–5 **B** sternites 10 and 11 **C** ultimate leg-bearing segment, ventral view **D** left coxopleural process, lateral view **E** ultimate leg, right lateral view. Scale bars: 1 mm (**A, B, D**); 2 mm (**C, E**).

**Figure 5.**
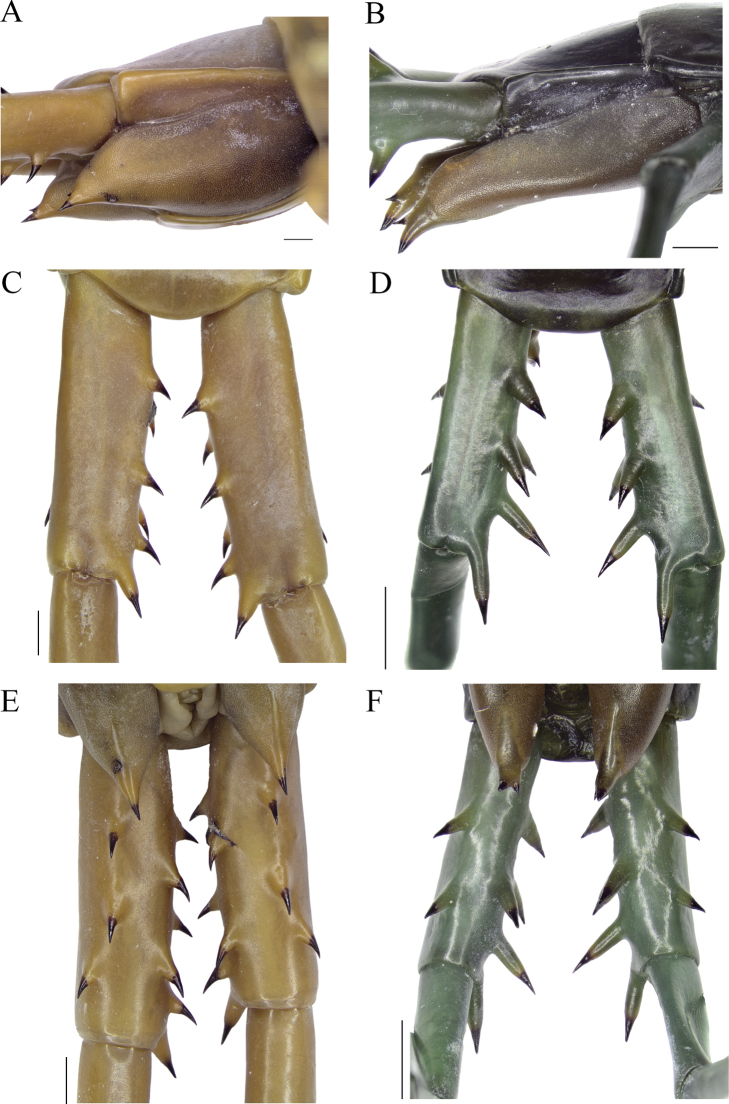
**A, C, E***Scolopendraspinosissima* Kraepelin, 1903; lectotype (ZMH-A0000633) **B, D, F***Scolopendraparadoxa* Doménech, 2018; holotype (CEUA017-Mr0000) **A, B** right coxopleura and coxopleural processes, lateral views **C, D** ultimate leg prefemora, dorsal views **E, F** ultimate leg prefemora, ventral views. The coxopleuron and coxopleural processes are different shapes and lengths; with the different sizes, morphologies, numbers, and positions of the prefemoral spinous processes, these are the most remarkable characters differentiating these species. Scale bars: 1 mm.

**Figure 6.**
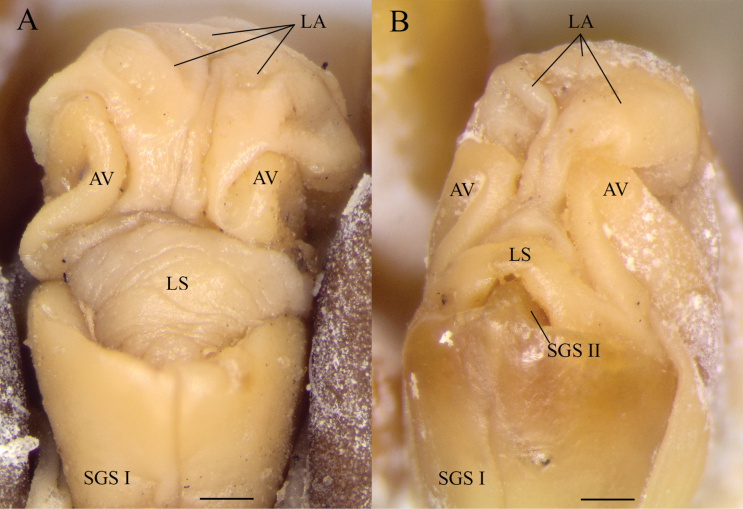
*Scolopendraspinosissima* Kraepelin, 1903; genital apparatus **A** paralectotype 2, female (ZMH-A00016059) **B** paralectotype 3, male (ZMH-A00016060). Abbreviations: AV = anal valve; LA = lamina adanalis; LS = lamina subanalis; SGS I = sternite of genital segment I; SGS II = sternite of genital segment II. Scale bars: 0.5 mm.

Figs 1 , 2 , 3 , 4 , 5A, C, E , 6 , 8E , Table 1

Scolopendra subspinipes var. spinosissima Kraepelin, 1903: 262–263.
Scolopendra spinosissima : Attems 1930: 31–32, fig. 44 (transcription, illustration; specimen unidentified); Lewis 2010: 92, 110, fig. 18 (in keys); Kronmüller and Lewis 2015: 269–278, fig. 2A (not type); Doménech et al. 2018: 401–427, table 4, figs 3A, B (not types), 4 (current paralectotype 3), and 15 (voucher, not type); Acuña et al. 2021: 417–419, figs 1, 2 (not type).

##### Diagnosis.

Colouration dark red to brownish. Antennae reaching posterior border of T3, rarely T4; with 19 antennal articles, basal four glabrous. Paramedian sutures on tergites highly variable in TT1–7, in TT8–20 complete. Paramedian sutures on sternite incomplete in SS 2–20. Free coxopleuron edge not extending beyond the T21 posterior edge. Coxopleural process moderately long and not inflected with coxopleuron, forming together an angle of ~ 120°. Coxopleural process with one AP and one smaller dorsal SAP, rarely with an extra ventral SAP. UL prefemur with single spine tipping long spinous processes disposed in VL: 1, V: 2, VM: 2, M: 1, DM: 2 and SP: 1. Penis, gonopods, and secondary sexual characters in males absent.

##### Lectotype

**(new designation).** Philippines • 1 unsexed adult; Luzon Island, Manila; 1902; H. W. Brölemann leg.; “ScolopendrasubspinipesLeach.var.spinosissima Krpln. ex Typis. Mus. Paris. [unreadable].VI.03. Manila”, “Scolop.subspinipesLeach.var.spinosissima Krpln. ex Typis. Mus. Paris. VI.03. Manila”, “*Scolopendrasubspinipesspinosissima* Kraepelin [red label fixed in jar]”; ZMH-A0000633. ***Paralectotypes*.** Philippines • 1 ♂, 1 ♀, 1 unsexed; Luzon Island, Quezón Province, Dolores; same collection data as for the lectotype; “Scolop.subspinipesLeach.var.spinosissima Krpln. Mus. Paris. 30.VI.03. Camarines. Philippinen”; ZMH-A00016058 to A00016060.

##### Type locality.

Since a lectotype is now designated, Manila, Luzon Island, Philippines (and not just Philippines) is the current type locality (Figs 1C, 2B, C) (ICZN 1999: Art. 73.2.3, 76.2; Kraepelin 1903).

##### Type depository

**(new data).** All type material is deposited in the collection of ZMH, Hamburg (Germany).

##### Legitum

**(new data).** H. W. Brölemann, 1902.

##### Distribution.

Philippines, endemic. Known from the islands of Luzon, Cebu, Negros, Guimaras, and Panay (Fig. 1C).

##### Current rank and status.

Accepted species.

##### Lectotype redescription

(variation of paralectotypes given in parentheses). Body length reaching 147 mm.

Live specimens dark red to brownish with cephalic plate and TT8–11 usually darker. Antennae and coxopleuron orange. Legs reddish to yellowish orange. Coxosternal surface and SS pale yellow (Fig. 1A, B).

Antennae reaching posterior border on T3 (T4 in paralectotype 1), with 19 articles (17–20 in paralectotypes 1 and 2), the basal four glabrous dorsally and ventrally (Fig. 3A). Cephalic plate with four ocelli in each side. Surface covered by dispersed small puncta allocating a short sensillum each; median sulcus absent. Posterior part of cephalic plate without paramedian sulci, overlapping the anterior margin of T1 (Fig. 3A). Coxosternite surface essentially smooth, counting with few isolated and less deep puncta; median suture absent (Fig. 3B, C). Article 2 of second maxillary telopodite with spur (Fig. 3C). Forcipula surface covered by dispersed small puncta. Left tarsungulum lost. Forcipular trochanteroprefemoral process with denticles in two groups, apically with two teeth on the right and three on the left, and proximally, one tooth on the right and two teeth on the left (a total of 2–5 in paralectotypes). Tooth-plates longer than wide, with small dispersed puncta and 7+8 teeth divided in two groups (5+5 in paralectotype 1). Tooth-plate with straight, transverse basal suture (Fig. 3C; Table 1).

**Table 1.** Morphological comparison between the type series of *Scolopendraspinosissima* Kraepelin, 1903, its voucher, and *S.paradoxa* Doménech, 2018 holotype. A = Absent; C = Complete; I = Incomplete; D = Distal; P = Proximal; PR = Partially retracted; R = Retracted. * = Appendix damaged. N/A = Not applicable.

	*Scolopendraparadoxa* Doménech, 2018	*Scolopendraspinosissima* Kraepelin, 1903				
Current type condition	Holotype –and voucher–	Lectotype	Paralectotype 1	Paralectotype 2	Paralectotype 3	Voucher (non type)
Specimen number	CEUA017-Mr0000	ZMH-A0000633	ZMH-A00016058	ZMH-A00016059	ZMH-A00016060	CEUA016-Mr0009
Reference in previous labels and catalogue	N/A	Mus. Paris c.; VI.03; “ex Typis” (holotype)	Mus. Paris c.; 30.VI.03; paratype	Mus. Paris c.; 30.VI.03; paratype	Mus. Paris c.; 30.VI.03; paratype	N/A
Body length in mm	132	147	126	120	99	87
Sex	R-Female	R	PR	Female	PR-Male	* (probably female)
Antenna reaching to tergite	T5	T3	T4	T3	T3	T4
Number of antennal articles	19/19	19/19	17/20	18/19	19/19	19/19
Number of proximal glabrous articles	4/4 dorsally 5½/5½ ventrally	4/4 dorsally and ventrally	4/4 dorsally and ventrally	4/4 dorsally and ventrally	4/4 dorsally and ventrally	4/4 dorsally and ventrally
Teeth on tooth-plate	5+5	7+8	5+5	7+7	7+7	7+7
Teeth on forcipular trochanteroprefemoral processes as total (upper group/lower group)	2 (1/1) – 1(1/0)	3 (2/1) – 5(3/2)	5 (3/2) – 3 (2/1)	2 (1/1) – 4 (3/1)	4 (3/1) – 4 (3/1)	4 (3/1) – 5(3/2)
Tergite paramedian sutures	TT1–2 A; TT3–4 IDP; T5C; T6C (right side ID); T7IDP; TT9–20C; T21 A	TT1–2 A; T3 IP; T4 C; T5 IP; T6 C; T7IDP; TT9–20C; T21 A	TT1A; TT2 ID; TT3–5 IDP; T6 C; TT7–8 IDP; TT9–20 C; T21 A	T1 A; T2 ID; T3 IDP; T4 C; T5 IDP; TT6–20C; T21 A	T1 A; T2 ID; T3 IDP; T4 C; T5 IDP; T6 C; TT7–20C; T21 A	TT1–2 A; T3 IP; T4–20 C; T21 A
First tergite with complete margination	10	10	12	10	10	12
Paramedian sutures on sternites	SS 1–2 A; SS3–18 C; SS19–21 A	S1 A; SS2–20 IDP; S21 A	S1 A; SS2–20 IDP; S21 A	S1 A; SS2–4 IP; SS5–19 IPD; SS20–21 A	S1 A; SS2–5 C; SS6–19 IPD; SS20–21 A	S1 A; SS2–12C; SS13–19 IP; S20–21 A
Spines in coxopleural process	AP: 1; SAP: 1	AP: 1; SAP: 1; (left with 1 extra ventral SAP spinula)	AP: 1; SAP: 1	AP: 1; SAP: 1	AP: 1; SAP: 1	AP: 1; SAP: 1
Coxopleural process extending beyond T21	Yes	No	No	No	No	No
Spinous process formula on prefemora of ultimate legs	VL: 2/2; V: 0/0; VM: 1/1; M: 1/1; DM: 2/2; SP: 1/1	VL: 1/1; V: 2/2; VM: 2/2; M: 1/1; DM: 2/2; SP: 1/1	VL: 1/1; V: 2/2; VM: 2/2; M: 1/1; DM: 2/2; SP: 1/1; (left M and VM proximal spinous processes with spines*)	VL: 1/1; V: 2/2; VM: 2/2; M: 1/1; DM: 2/2; SP: 1/1	VL: 1/1; V: 2/2; VM: 2/2; M: 1/1; DM: 2/2; SP: 1/1; (right VM proximal spinous process hardly noticeable, without spine); (Doménech et al. 2018: fig. 4A–C)	VL: 1/1; V: 2/2; VM: 2/2; M: 1/1; DM: 2/2; SP: 1/1; (left VL with a small medial extra aberrant process)
Legs with one tarsal spur	1–18 (left leg 18*)	1–20 (right leg 19)	1–20	1–20 (several mid body legs *)	1–20	1–20 (right leg 19)

Spiracles positioned in segments 3, 5, 8, 10, 12, 14, 16, 18 and 20, triangular in form and tri-valved (Fig. 3D). Tergite surface with shallow, small, and more dispersed puncta compared to the cephalic plate (Fig. 4A). Paramedian sutures of tergites faint and variable; in paralectotype sutures on T1 and T21 absent, T2 incomplete posteriorly, T3, T5, and T7 incomplete posteriorly and anteriorly, T4, T6, and TT8–20 complete (see Table 1 for paralectotypes). Complete margination starting on T10 (on T12 in paralectotype 1). Tergite of ultimate leg-bearing segment with disperse and non-deep puncta, without depression or sutures; posterior margin rounded. Ratio of width:length of tergite of ultimate leg-bearing segment 1.14:1.Sternite surfaces essentially smooth, with dispersed, small, shallow puncta. Paramedian sutures in S1 and S21 absent; in SS2–20 incomplete and confined to proximal 10–25% and distal 5–10% of sternite length (see Table 1 for paralectotypes; Fig. 4B). Space between sutures sometimes weakly depressed. Sternite of ultimate leg-bearing segment with sides converging posteriorly (Fig. 4C); surface without depressions or sutures.

Coxopleuron with numerous coxal pores; reaching but excluding spines of the coxopleural process, not extending beyond T21 posterior margin of T21. Free edge on coxopleuron moderately long, with straight dorsal and ventral margins. Posterodorsal margin of coxopleuron not inflected into dorsal margin of coxopleural process, forming both margins at ~ 120° angle (Figs 4C, D, 5A). Coxopleural process moderately long, with isolated small pores and with two or three distal spines, two on right (one each AP and smaller dorsal SAP) and three on left (an additional minute spine in ventral SAP; Fig. 4D). Lateral or dorsal spines absent. Pore-free area extending ventrally 30% of length from proximal part of coxopleural process to margin of sternite of the ultimate leg-bearing segment (Figs 4C, 5A).

All legs without tibial spurs. Surface with shallow, dispersed, small puncta allocating a short sensillum each. One tarsal spur on legs 1–19 or 20, right and left legs, respectively (all paralectotypes with spur on legs 1–20). UL long, slender, with length ratios prefemur and femur = 1.2:1, femur and tibia = 1.07:1, tibia and tarsus 2 = 2:1; tarsus 1 and tarsus 2 = 1.45:1 (Fig. 4E). Prefemora flattened dorsally, with long wider base processes located backwards at 45° angle with respect to the prefemur. Spines of the spinous processes slightly curved backwards. Prefemoral spinous processes formula: VL: 1, V: 2, VM: 2, M: 1, DM: 2 (Figs 4E, 5C, E), (in paralectotype 3, proximal spine in VM position in right prefemur is absent (preserving the prefemoral process); Doménech et al. 2018: fig. 4). Prefemoral corner process slightly longer and with a narrow base in respect to other prefemoral processes, ending with a single non-curved spine (Figs 4E, 5C, E). Tarsus 1 partially lost in left UL (Fig. 4E).

Genitalia in the lectotype and paralectotype 1 retracted. In paralectotypes 2 and 3 well-developed (Fig. 6A, B, respectively), partially retracted, reaching further than the distance between posterior margin of ULBS sternite and distal part of the coxopleural process. The genital segment sternite 1 rounded, convex posteriorly, with a median suture. Tergite of the genital segment without small setae. In male paralectotype 3 (Fig. 6B) genital segment 2 is small, horseshoe-shaped, with small shallow puncta; penis, gonopods, and secondary sexual characters absent.

### ﻿*Scolopendraspinosissima* differential diagnosis (*S.spinosissima* paratypes features given in parentheses)

**Figure 7.**
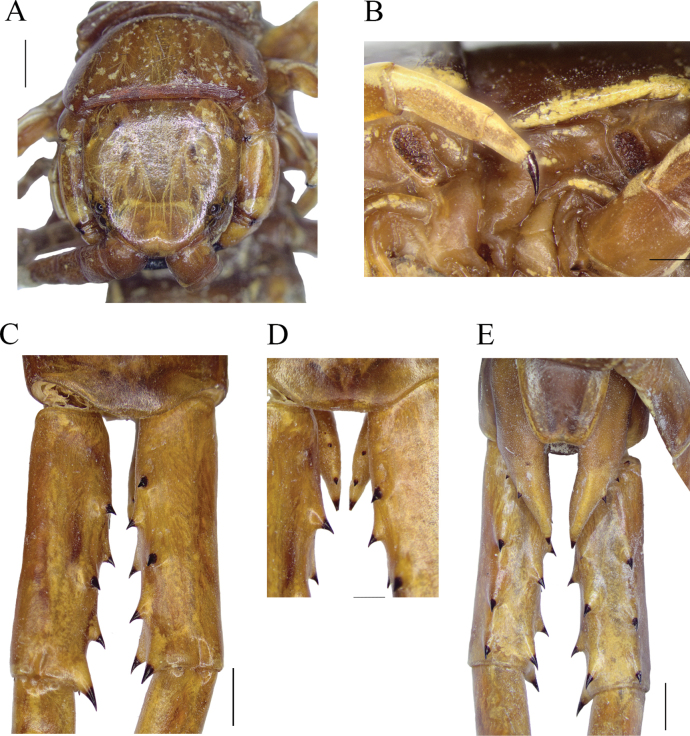
*Ethmostigmusrubripesrubripes* (Brandt, 1840) (ZMH-A00016061). Previously included in the type series of *S.spinosissima* under the label “Mus. Paris I.XI.03” **A** cephalic plate and tergite 1, dorsal view **B** left spiracles on segments 7 and 8 **C**UL prefemur, dorsal view **D** coxopleuron, dorsal view **E** ultimate leg-bearing segments, coxopleuron and ultimate leg prefemora, ventral view. Scale bars: 0.5 mm (**B, D**); 1 mm (**A, C, E**).

**Figure 8.**
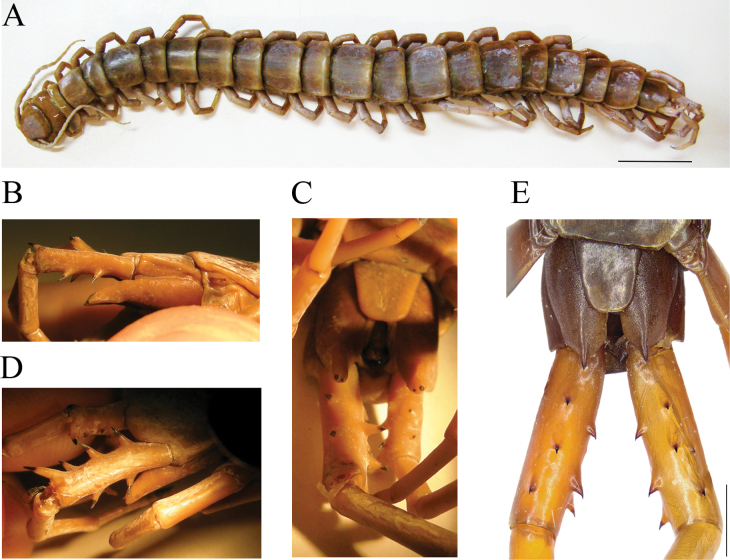
**A–D***Scolopendraparadoxa* Doménech, 2018; non-type (MNHN N° 387), identified by Kraepelin (1904b) as *S.spinosissima* (total length 176 mm) **E***S.spinosissima*; non-type (CEUA016-Mr0009, voucher) **A** habitus, dorsal view **B** coxopleuron and ultimate legs prefemur, right lateral view **C** ultimate leg-bearing segment and ultimate legs prefemur, ventral view **D** right ultimate leg prefemoral spinous processes (left ultimate leg regenerated), dorsolateral view **E** ultimate leg-bearing segment and ultimate legs prefemora, ventral view. Notice the smaller extra aberrant median VL spinous process in the left prefemur observed exclusively in this specimen. Scale bars: 1 mm (**E**); 20 mm (**A**).

According to Doménech et al. (2018), and re-examination of both type series, the closest relative to *S.spinosissima* is *S.paradoxa.* This species can be readily distinguished from *S.spinosissima* on the basis of the following unambiguous characters: 1) antennae usually reaching T5 (vs T3, rarely T4); 2) first four basal antennal articles glabrous dorsally and first 5–5½ glabrous ventrally (vs four basal glabrous over all their surfaces); 3) cephalic plate surface covered only anteriorly by disperse small puncta (vs whole cephalic plate covered by small sparse puncta); 4) coxopleuron free edge very long, clearly extending beyond the posterior edge of T21 (vs coxopleuron reaching but not extending beyond T21) (Fig. 5A, B); 5) posterodorsal margin of coxopleuron forming an angle of ~ 180° angle with dorsal margin of coxopleural process (vs ~ 120° in *S.spinosissima*) (Fig. 5A, B); 6) coxopleural process elongate and large, with two spines (vs short and smaller, with two or rarely three spines) (Fig. 5A, B); 7) UL prefemoral spinous with seven, extremely long, narrow base processes; spines almost straight, consistently two in VL position, zero in V position, and just one in VM position (vs nine distinctly shorter spinous processes with a wide base, and with spines slightly curved backwards, constantly with a single distal process in VL and with two each in V and VM positions) (Fig. 5C–F); 8) legs with one tarsal spur in 1–18, rarely 1–19 (vs generally 1–20); 9) evident aposematic colouration, with orange antennae, dichromatic tergites orange, yellowish, or dark green anteriorly, and dark blue or brown posteriorly, cyan legs (vs not so pronounced aposematic colouration, with monochromatic brownish or dark red tergites, and with orange antennae and legs); 10) size up to 176 mm (vs up to 147 mm); 11) semiaquatic behaviour (vs exclusive terrestrial lifestyle) and 12) partial cytochrome *c* oxidase (COI) sequences genetic distances between 18.2–19.6% (Doménech et al. 2018).

Moreover, *S.spinosissima* as well as *S.paradoxa*, can be differentiated from all remaining Southeast Asian congeners, but also from all species in the genus *Scolopendra* by the exclusive discontinued paramedian sutures on TT and SS (Table 1), and by the unique shape, size, and disposition of the UL spinous processes (Doménech et al. 2018).

### ﻿Fixation of *Scolopendraspinosissima* specimens making up the type series

On 15 December 1903, Karl Kraepelin’s paper describing *S.spinosissima* was published, lacking the type series designation or an explicit depository. These specimens were indispensable for the detailed morphological comparison with *S.paradoxa* types and to confirm the taxonomic identity of *S.spinosissima* specimen molecularly analysed by Doménech et al. (2018). When a type series is not designated, the ICZN allows the use of other information sources besides that provided in the original description paper to ensure which specimens comprise such type series (ICZN 1999: Art. 72.4.1.1).

In the original work, Kraepelin (1903) indicated that the *S.spinosissima* specimens he examined [= type series] had been previously stored at the Natural History National Museum of Paris (MNHN) with the abbreviation "Mus. Paris", without stating these samples’ definitive depository. Otherwise, several authors (Weidner 1960; Rack 1974; Doménech et al. 2018; Thofern et al. 2021) showed the probable presence of these specimens at the Natural History Museum of Hamburg (ZHM), the place where Kraepelin mainly developed his career by studying his own and exchanged material (Lohmann 1915; Harms and Dupérré 2018; Monod et al. 2019; Thofern et al. 2021). At the ZHM, a total of five S.subspinipesvar.spinosissima specimens were found. In their labels and collection data, all samples were mentioned as in the original publication with the identical provenance inscription “Mus. Paris” (Fig. 2A, B). Two of these labels (top labels in Fig. 2B) indicate type material of one of the specimens by using the term “ex Typis” (meaning “coming from the type series” sensu Harms and Dupérré 2018; Monod et al. 2019; Thofern et al. 2021; N. Dupérré pers. comm. Jun. 2021). By ‘adding to’ the label information, the ZHM catalogue provided more collection data and clearly shows the paratype status of the four remaining specimens (Fig. 2C). At the same time, this catalogue also demonstrated the direct connection between the specimens and the 1903 publication, with reference to the pages where this species was originally described (“*S.*” German abbrev. of “Seiten”, pages; pp 262–263; Fig. 2A–C). Kraepelin’s calligraphy on all these documents also matches with the one seen in modern literature (Harms and Dupérré 2018; Monod et al. 2019; Thofern et al. 2021). The additional information in the Weidner file cards and current ZHM catalogues are consistent with these findings (Weidner 1960; Rack 1974; Thofern et al. 2021).

Finally, once it was established that no information supported the inclusion of the other *S.spinosissima* specimens known by Kraepelin (1904b; see justification below), the entire data set provided here (ICZN 1999: Art. 72.4.7) revealed that the original description of *S.spinosissima* was based only on five specimens deposited at the ZHM collection. After morphological analysis of the five individuals originally used by the species' authority, only four of them, here fixed, make up the type series of *S.spinosissima* (ICZN 1999: Art. 72.1.1). These are the one labelled as “Mus. Paris. VI.03 ex Typis” and three labelled as “Mus. Paris. 30.VI.03” but excluding the specimen “Mus. Paris. I.IX.03” as this one does not satisfy the new proposed morphological criteria for *S.spinosissima* (see below).

### ﻿*Scolopendraspinosissima* lectotype designation

The ICZN (1999) only allows a holotype designation in two circumstances: the express designation in the original publication or by monotypy (Art. 73.1.1, 73.1.4, 73.1.2, Recommendation 73F). Therefore, the type designation made by Kraepelin in the catalogue or labels (Fig. 2B, C) becomes nomenclaturally invalid since these do not constitute enough evidence of the fixation of a specimen as a type in the sense of ‘The Code’ (ICZN 1999: Art. 8, 9.8, 72.4.7, 73.1). Subsequently, all five *S.spinosissima* specimens were considered syntypes (ICZN 1999: Art. 72.1.1). However, in the interest of nomenclatural stability and with the aim of clarifying the application of the name for the taxon *S.spinosissima*, the lectotype designation (ICZN 1999: Recommendation 73F, Art. 74.7) proved justified to solve the following nomenclatural conflicts: 1) presence of two different taxa in the original *S.spinosissima* type series and 2) the need for a reference specimen for its clear morphological redefinition and, as a consequence, unambiguous differentiation in respect to its closest relative *S.paradoxa* (ICZN 1999: Preamble, Art. 13, Recommendation 73F, 74G, Art. 74, 74.7.3; Doménech et al. 2018). Kraepelin did not validly designate a holotype among the specimens making up the *S.spinosissima* type series (ICZN 1999: Art. 72.4.7, 73.1). Nonetheless, he provided sufficient evidence supporting one of these types: after reviewing the catalogue annotations where all specimens in the type series, with except of one, were [invalidly] regarded as paratypes (see in Fig. 2C the term “Paratyp” and the red line dividing the species in two groups). Therefore, the holotype being the counterpart of this term (ICZN 1999: Art. 72.4.5) and in the absence of any additional data corroborating the existence of other types (see below), it can be inferred that Kraepelin may have selected the specimen “Mus. Paris. VI.03 ex Typis”, to be deemed the benchmark of *S.spinosissima.* In line with this, based on the ZMH catalogue information (Fig. 2C), a unique red label stamped into the “Mus. Paris. VI.03 ex Typis” jar was found (Fig. 2A). Red colours on labels usually indicate the type status of a specimen (Calhoun and Hawkins 2016) but when this colour appears in a single label, it often points to the name-bearing type of a species (Ratcliffe 2013). Hence, in the absence of any other red labels, this is advocated as the author’s recognition of this specimen as the reference for *S.spinosissima.* Consequently, the best developed and preserved adult syntype (“Mus. Paris. VI.03 ex Typis”) is designated as lectotype, and therefore, as the name-bearing type for the species (Figs 2–4, 5A, C, E; ICZN 1999: Art. 73, 74). Any lectotype designation relegates the remainder of the type series as paralectotypes (ICZN 1999: Art. 74.1.3). Subsequently, the three specimens contained in the jar “Mus. Paris. 30.VI.03” now bear the status of paralectotypes of *S.spinosissima* (Fig. 2B, C, Table 1). The previous label of the “Mus. Paris. VI.03 ex Typis” specimen as “paratypoid” (Weidner 1960; Weidner file cards; Rack 1974) or more precisely as syntype (ICZN 1999: Art. 72.1.1, 74.1.3; Thofern et al. 2021) is now substituted by the current lectotype status (ICZN 1999: Art. 74.1.2).

With regards to the previously illustrated paralectotype 3 in Doménech et al. (2018: fig. 4) and the recommendation of clause 74B (ICZN 1999) of “Preference for illustrated specimen”, it is concluded that this specimen is not eligible over the newly designated lectotype since the former specimen does not fulfil the premise of “Other things being equal”, due to its subadult developmental stage, partially aberrant prefemoral processes of the UL, and Kraepelin’s express designation of this specimen as a support specimen, naming it as paratype (Table 1; Fig. 2B, C; Doménech et al. 2018: fig. 4). According to this recommendation, the specimen illustrated by Attems (1930) could not be considered because it was not designated as a type nor was a determinate taxon ascribed to such an illustration (ICZN 1999: Art. 74, Recommendation 74B).

### ﻿Exclusion of the Mus. Paris. I.XI.03 specimen from the type series

The “Mus. Paris. I.IX.03” specimen displays four ocelli on each side of the cephalic plate, overlapped by T1 (Fig. 7A), ten pairs of non-valved, round, or oval spiracles on segments 3, 5, 7, 8, 10, 12, 14, 16, 18, and 20, the first one being the largest (Fig. 7B), forcipular trochanteroprefemoral process absent, smooth tergites, and coxopleural process with spines in apical, dorsal, and lateral positions. All these characters place this specimen within the genus *Ethmostigmus* Pocock, 1898 (Joshi and Edgecombe 2018; Schileyko et al. 2020). In the Philippines, the genus *Ethmostigmus* is only represented by *E.rubripesplatycephalus* (Newport, 1844), was only documented in the Spratly Islands (South China Sea; Schileyko and Stagl 2004; Schileyko and Stoev 2016). The current identification represents the second formal record for the genus in this archipelago, from Mindanao Island in the Philippines (Fig. 2B). The specimen “Mus. Paris. I.IX.03” shows a relatively long coxopleural process, with one apical, one subapical and two lateral spines, and a slightly arcuate edge with two or three spines (identical features to the specimen from the Moluccas Islands (Indonesia), presented in Schileyko and Stagl (2004: fig. 33–35). This specimen also has 18 antennal articles (first basal four dorsally glabrous), tooth-plate with 3+3 teeth, complete paramedian sulci on TT3–20, weak paramedian sulci on SS3–20, and the prefemoral spinous process formula (here revised) VL: 3, V: 0, VM: 2 (3 on the left), M: 2, DM: 2 and SP: 1 (Fig. 7C, D), all of which supports its identity as *E.rubripesplatycephalus* sensu Schileyko and Stagl (2004).

*Ethmostigmusrubripesplatycephalus* mainly differs from *E.rubripesrubripes* (Brandt, 1840) in the lengths of the coxopleural processes, which in the first is more than twice as long as S21 (Attems 1930; Schileyko and Stagl 2004). As shown in Kraepelin’s drawing (1903: fig. 107) and the illustration of one of the syntypes (Joshi and Edgecombe 2018: fig. 2C, E, G, H), the two reduced coxopleuron lateral spines, almost conical morphology, and the coxopleural process being greater in length in *E.rubripesplatycephalus* are features that are obviously not present in the specimen “Mus. Paris. I.IX.03”. On the basis of these morphological characters, and despite the fact that this subspecies has not been reported in the Philippines, the specimen *S.spinosissima* “Mus. Paris. I.IX.03” is here identified as *Ethmostigmusrubripesrubripes*, and therefore excluded from the type series of *S.spinosissima* (ICZN 1999: Recommendation 72B).

### ﻿Non-inclusion of the MNHN*S.spinosissima* specimens as part of the type series

In addition to the material stored at the ZMH collection (Kraepelin 1903; Fig. 2A–C), Kraepelin (1904b) reported the presence of additional specimens of *S.spinosissima* at the MNHN collection. In that author’s catalogue, three sets of *S.spinosissima* specimens were classified in a list according to their locality, namely: specimen/s from Luzon, det. 1875 by Laglaize [samples lost]; Jar N° 388 comprising ten specimens from Dolores, Tayabas Province (currently Quezon) [placed erroneously in the Camarines Peninsula]; and Jar N° 387, containing one specimen from Manila. The specimens in the two jars were also identified as *S.multidens* Newport, 1844 by Eugène Simon prior to Kraepelin’s work (1904b) (see Materials and methods section). Morphologically, the ten specimens in jar N°388 fit with the redescription of *S.spinosissima* type series. The additional specimen in jar N°387 is confirmed as *S.paradoxa* due to the following morphological traits: antennae reaching T5, 19 antennal articles (right antenna damaged), first basal four dorsally and 5½ ventrally glabrous; punctation only in the anterior part of cephalic plate; legs 1–18 with one tarsal spur; free edge of the coxopleuron long, extending beyond the T21 with a large coxopleural process ending with two spines; and finally, the right UL (left UL regenerated) with the seven diagnostic, extremely long spinous processes, tipped with an almost straight spine, disposed in the regular prefemoral formula for this species (VL: 2, V: 0, VM: 1, M: 1, DM 2 and SP: 1; Fig. 8B–D).

Despite Kraepelin’s knowledge of these eleven specimens in the MNHN collection (Kraepelin 1904b), no evidence was found to suggest that these samples were used for the original description of *S.spinosissima* (Kraepelin 1903; ICZN 1999: Art. 72.4.1.1). In his MNHN catalogue from 1904, Kraepelin (1904b) did not mention the type status of any *S.spinosissima* specimens stored there. On the contrary, the terms “Type”, “Types!”, and “Co-types!” were clearly ascribed to other specimens in those publications (Kraepelin 1904a, b). Some of these samples were components of a type series divided between the MNHN and ZMH collections (see Kraepelin 1904a, b; Thofern et al. 2021), but again, there was no evidence of this for *S.spinosissima.* Regarding Kraepelin’s labels, he did not mention the type status of the MNHN*S.spinosissima* specimens, but again, indicated this status in the labels of other specimens belonging to species described by himself or other authors (Kraepelin 1903, 1904a, b; Doménech 2024). Therefore, this lack of explicit designation can only be understood as an intentional act by Kraepelin’s of non-declaration of *S.spinosissima* specimens from the MNHN collection as part of the type series.

In addition to this absence of explicit designations, four other relevant considerations for not including of the MNHN specimens in the type series of *S.spinosissima* are as follows:

1. different publication year involving other MNHN specimens (1904, a year after the original description of *S.spinosissima* which was based exclusively on animals stored in Hamburg (Kraepelin 1903, 1904a, b; ICZN 1999: Art. 72.4.1.1);
2. Kraepelin’s reference to himself in the text as *S.subspinipes* var. *spinosissma*Kraepelin [1903] in his 1904b study is impossible as there was no previous work describing the species; ICZN 1999: Chapters 3–6);
3. incompatibility between the size of the species detailed in the original description versus those of the MNHN specimens (up to 150 mm [actual 146] vs up to 176 mm) proving that Kraepelin did not use these much larger specimens in his 1903 description (see Table 1; Fig. 8A; ICZN 1999: Art. 72.4.1);
4. the presence of several Kraepelin documents confirming the exclusive type status of the ZMH specimens (Fig. 2A–C; Kraepelin 1903).

Therefore, in the absence of evidence and in the presence of other data arguing against inclusion, the consideration of the specimens from MNHN collection as part of the type series of *S.spinosissima* (Thofern et al. 2021) is finally discarded.

### ﻿Remarks on the *S.spinosissima* type series collector, depositories history, and type locality

In 1903, Karl Kraepelin described this taxon on the basis of specimens stored at the ZMH, which were previously loaned by the MNHN collection (Kraepelin 1903; Weidner 1960; Rack 1974; Thofern et al. 2021; see also ZMH file cards and Fig. 2B, C). However, in the MNHN, non-direct data involving the collector of *S.spinosissima* type series was located. In 1904, Kraepelin also observed, labelled, and registered the presence of other *S.spinosissima* specimens at the MNHM collection (see above; Kraepelin 1904b). Despite the fact that his catalogue was more accurate than his specimen labels, Kraepelin provided identical localities for almost all specimens deposited in Paris and in Hamburg (Fig. 2B, C; Kraepelin 1903, 1904b). Additionally, the specimens in the Paris collection date back to 1902. According to Kraepelin (1904b), the type series from Hamburg (“Mus. Paris”) and date of description (1903) offered chronological compatibility, which suggests that the specimens from the ZMH and MNHN collections were collected simultaneously by the same collector. All this points to H. W. Brölemann, a taxonomist with confirmed participation in an expedition to the Philippines in 1902 (see Kraepelin 1904a, b). Therefore, it is probable that this collector deposited some *S.spinosissima* specimens in the MNHN collection and permanently loaned the remaining ones (making up the type series) to the ZMH collection (Weidner 1960; Rack 1974; Doménech et al. 2018; Thofern et al. 2021).

The previous type locality of *S.spinosissima* was simply detailed as Philippines by Kraepelin (1903). However, according to the ZMH collection data (Fig. 2A–C), the lectotype and hence the type locality are now bound to Manila, while the paralectotype locality is placed in the Camarines area. Despite lack of confirmation, this ZMH information, in combination with the previous MNHN data (Kraepelin 1903, 1904b; see also Examined material), suggests that the paralectotypes are also from Dolores, Tayabas Province (currently in Quezon), and not from somewhere in the Camarines Peninsula where no other Dolores exists, or existed (Fig. 1C).

### ﻿Taxonomy of *S.spinosissima* voucher and use of its DNA barcode

In its integrative work, Doménech et al. (2018) analysed the COI partial sequence of the specimen CEUA016-Mr0009, which was identified as *S.spinosissima* (Table 1, Fig. 8E; but see also Doménech et al. 2018: fig. 15; Buckner et al. 2021). When this voucher was compared with the types of *S.paradoxa*, the morphological and molecular features showed that these specimens belong to two distinct taxa (Doménech et al. 2018). In this work, the morphology of this specimen was compared with the *S.spinosissima* lectotype, and both specimens were found to be conspecific (Table 1; Figs 5E, 8E). This confirms that the previous identification of the voucher of *S.spinosissima* was correct and is also resolved in the clear molecular and taxonomic separation of *S.spinosissima* and *S.paradoxa*.

### ﻿Revised key for the species of *Scolopendra* from Philippines

Note: Due to insufficient data, *S.multidens* Newport, 1844 is excluded from the Philippines faunal catalogue until new evidence confirms its presence in the archipelago (see below).

**Table:** 

1	4 basal antennal articles glabrous dorsally	**2**
–	6 or more basal antennal articles glabrous dorsally	**3**
2	Coxopleuron not clearly extending beyond the T21 posterior edge. Prefemoral formula: VL: 1, V: 2, VM: 2, M: 1, DM: 2, SP: 1	***S.spinosissima* Kraepelin, 1903**
–	Coxopleuron clearly extending beyond the T21 posterior edge. Prefemoral formula: VL: 2, V: 0, VM: 1, M: 1, DM: 2, SP: 1	***S.paradoxa* Doménech, 2018**
3	T21 with median suture	***S.morsitans* Linnaeus, 1758**
–	T21 without median suture	**4**
4	Prefemoral formula VL: 2, V: 0, VM: 0, M: 2 (1), DM: 2 (1), SP: 2. Coxopleural process with 2 AP plus 0–1 SAP spines	***S.subspinipes* Leach, 1816**
–	Prefemoral formula VL: 3, V: 3, VM: 2, M: 2, DM: 2, SP: 4–6. Coxopleural process with 4, rarely with 3 AP spines	***S.subcrustalis* Kronmüller, 2009**

## ﻿Discussion

The designation of the type series of *S.spinosissima* was necessary to clarify the morphological boundaries of this species, and the identities of the types and vouchers of *S.spinosissima* and *S.paradoxa*. Apart from the more precise type locality, the major improvement of this *S.spinosissima* redescription is the revised UL prefemoral processes formula. This is now more precise (i.e., V: 2), substituting the previously inaccurate range-based formula (i.e., V: 2–3). Also the position of one of the spinous processes, previously placed in the V position, was clarified as actually being on the distal VL. Other relevant morphological features added are the length, shape, and relative position of the coxopleuron in respect of the coxopleural process, the number of legs with a tarsal spur, the presence of the SS paramedian sulci, and descriptions of antennal setae distribution, tegument punctuation, and spinulation variations of the coxopleural processes.

Another important progress was the comparative morphological analysis of the voucher specimen CEUA016-Mr0009 of *S.spinosissima*, for which *S.spinosissima* sensu Doménech et al. (2018) was confirmed as *S.spinosissima* sensu Kraepelin (1903). This demonstrated that *S.paradoxa* was not described before 2018. All those facts support the previous morphological, molecular, and taxonomic outcomes (Doménech et al. 2018) for these now clearly and objectively separated species.

Repeated misidentifications of the historical specimens of *S.spinosissima* as *S.multidens* (see Examined materials), a species with only a single old citation from Mindanao (Wang 1962) (Fig. 2B), suggest that the presence of this taxon in the Philippines should be reconsidered. *Scolopendramultidens* has only been reported from southwest continental Asia, Java (Indonesia), and doubtfully from New Guinea (Bonato et al. 2016; Siriwut et al. 2015, 2016). The taxonomic re-evaluation of the specimens reported by Wang (1962) and new samples from Mindanao, New Guinea, and nearby areas could solve all these questions.

Finally, this study also revealed two distinct taxonomic criteria for the identification of the two *E.rubripes* subspecies (compare Schileyko and Stagl 2004 with Joshi and Edgecombe 2018). Following the preference for the depicted syntype (Joshi and Edgecombe 2018), the presence of *E.rubripesplatycephalus* in the Philippines (Schileyko and Stagl 2004; Schileyko and Stoev 2016) needs a re-evaluation and the specimen from the Spratly Islands (Schileyko and Stagl 2004) should be assigned to *E.rubripesrubripes*. Due to the morphological variability also observed between *E.rubripes* s. str. (Schileyko and Stagl 2004) and the two currently accepted subspecies, these taxa require new standardized diagnostic criteria and re-examination of taxonomic rank for the considerable distinct subspecies *E.rubripesspinosus* (Newport, 1844).

This taxonomic assessment of two species of *Scolopendra* is a primary step towards increasing biodiversity knowledge and developing conservation strategies involving these venomous arthropods with potential for agricultural and pharmaceutical applications.

## Supplementary Material

XML Treatment for Scolopendra spinosissima

## ﻿Additional information

### Author ORCIDs

Carles Doménech https://orcid.org/0000-0003-1890-9434

### Data availability

All of the data that support the findings of this study are available in the main text.
